# The Effects of Non-Invasive Brain Stimulation on Quantitative EEG in Patients With Parkinson's Disease: A Systematic Scoping Review

**DOI:** 10.3389/fneur.2022.758452

**Published:** 2022-03-02

**Authors:** Thaísa Dias de Carvalho Costa, Clécio Godeiro Júnior, Rodrigo Alencar e Silva, Silmara Freitas dos Santos, Daniel Gomes da Silva Machado, Suellen Marinho Andrade

**Affiliations:** ^1^Aging and Neuroscience Laboratory, Universidade Federal da Paraíba, João Pessoa, Brazil; ^2^Division of Neurology, Hospital Universitario Onofre Lopes, Universidade Federal do Rio Grande do Norte, Natal, Brazil; ^3^Department of Physical Education, Universidade Federal do Rio Grande do Norte, Natal, Brazil

**Keywords:** electroencephalography, transcranial direct current stimulation (tDCS), repetitive transcranial magnetic stimulation (TMS), transcranial alternating current stimulation (tACS), non-invasive brain stimulation (NIBS), Parkinson's disease

## Abstract

Parkinson's disease (PD) is a progressive neurodegenerative disorder characterized by motor and non-motor symptoms, aside from alterations in the electroencephalogram (EEG) already registered. Non-invasive brain stimulation (NIBS) techniques have been suggested as an alternative rehabilitative therapy, but the neurophysiological changes associated with these techniques are still unclear. We aimed to identify the nature and extent of research evidence on the effects of NIBS techniques in the cortical activity measured by EEG in patients with PD. A systematic scoping review was configured by gathering evidence on the following bases: PubMed (MEDLINE), PsycINFO, ScienceDirect, Web of Science, and cumulative index to nursing & allied health (CINAHL). We included clinical trials with patients with PD treated with NIBS and evaluated by EEG pre-intervention and post-intervention. We used the criteria of Downs and Black to evaluate the quality of the studies. Repetitive transcranial magnetic stimulation (TMS), transcranial electrical stimulation (tES), electrical vestibular stimulation, and binaural beats (BBs) are non-invasive stimulation techniques used to treat cognitive and motor impairment in PD. This systematic scoping review found that the current evidence suggests that NIBS could change quantitative EEG in patients with PD. However, considering that the quality of the studies varied from poor to excellent, the low number of studies, variability in NIBS intervention, and quantitative EEG measures, we are not yet able to use the EEG outcomes to predict the cognitive and motor treatment response after brain stimulation. Based on our findings, we recommend additional research efforts to validate EEG as a biomarker in non-invasive brain stimulation trials in PD.

## Introduction

Parkinson's disease (PD) is a progressive neurodegenerative disorder caused by the degeneration of the dopaminergic neurons of the substance nigra pars compacta and involvement of other neural circuits, resulting in motor and non-motor symptoms (1, 2). Although medicinal therapy and deep brain stimulation (DBS) can be chosen as the treatments for these patients, non-invasive brain stimulation (NIBS) techniques have been suggested as an alternative therapy with related rehabilitative effects (3–6).

The most used NIBS techniques for motor and cognitive rehabilitation are transcranial magnetic stimulation (TMS) and transcranial electrical stimulation (tES), which include transcranial direct stimulation (tDCS) and transcranial alternating current stimulation (tACS) (7). Despite the benefits associated with the use of NIBS in the treatment of patients with PD, such as the improvement of motor (3, 5, 8) and non-motor (9–11), the neurophysiological changes associated with these techniques are still unclear. In this regard, the electroencephalogram (EEG) is a tool of interest due to the possibility of identifying the changes in bioelectrical brain activity, which presents as a potential neurophysiological biomarker and prognosis for clinical management of PD (12, 13).

Studies with EEG in patients with PD have shown an excessive coherence of the beta frequency related to the motor symptoms (14, 15), while other studies showed low dominant frequencies or increased spectral power of lower frequencies bands, which are related to cognitive impairment (12, 16). NIBS can modify the cerebral oscillations and their associated functions, such as increased synchronization of the frequency bands of the EEG (17), decrease the spectral power of low or high frequencies (18, 19), suggesting a possible link between beta and gamma frequencies with the anti-kinetic and prokinetic effects, respectively (20). Finally, a review concluded that the modulation of beta frequency may be a consolidated marker of the success of NIBS in PD, however, it presented only preliminary results from TMS and tACS (21).

Nonetheless, despite studies that have investigated the effects of NIBS intervention on EEG oscillations, the variety of NIBS techniques and protocols and the different conditions in which the EEG was measured may lead to confusion in interpretation and future directions. Therefore, we conducted a systematic scoping review aiming to identify the nature and extent of research evidence on the effects of NIBS on the cortical activity measured by the EEG in patients with PD. Beyond presenting a summary of the body of available evidence, we will highlight existing gaps in the literature and discuss the possible paths for conducting future studies.

## Methods

The current study consisted of a systematic scoping review (22, 23), conducted and reported according to the guidelines of the *Preferred Reporting Items for Systematic reviews and Meta-Analyses extension for Scoping Reviews (PRISMA-ScR)* (24). The review process was performed using the Rayyan platform (25), developed by the Qatar Computing Research Institute. The protocol of the revision was registered in the Open Science Framework (https://osf.io/2zvs3/).

The search strategy was configured by gathering evidence, without language restriction, from inception until April 2020, on the following basis: PubMed (MEDLINE), PsycINFO, ScienceDirect, Web of Science, and cumulative index to nursing & allied health (CINAHL). The following search terms, with the Boolean operators AND/OR, were used: “Parkinson disease”; “Parkinson's disease; “electroencephalography”; “electroencephalogram”; “EEG”; “transcranial direct current stimulation”; “tDCS”; “transcranial magnetic stimulation”; “TMS”; “non-invasive brain stimulation”; “NIBS”; “transcranial electrical stimulation”; “binaural beats (BBs)”; “galvanic vestibular stimulation (GVS)”; “transcranial alternating current stimulation”; and “tACS.” The strategy was adjusted for each database following the example of PsycINFO (Table 1).

**Table 1:** Search strategy for PsycINFO database.

(“Parkinson disease” OR “Parkinson's disease”) AND (electroencephalography OR EEG) AND (“transcranial direct current stimulation” OR tDCS OR “binaural beats” OR “galvanic vestibular stimulation” OR tACS OR “transcranial magnetic stimulation” OR “non-invasive brain stimulation”).

*EEG, electroencephalogram; tDCS, transcranial direct current stimulation; tACS, transcranial alternating current stimulation*.

The inclusion criteria for the selection of studies were as follows: (1) enroll participants diagnosed with idiopathic PD; (2) perform any type of NIBS as the intervention; (3) present quantitative EEG as the pre-intervention and post-intervention outcome measures; and (4) to be a clinical trial. Case studies, simulations studies, conference abstracts, studies that used the NIBS for diagnoses purposes or used the EEG only for safety reasons (i.e., identification of epileptic waveforms) were excluded.

After removing the duplicates, two independent reviewers screened the results of the searches based on the titles and abstracts and applied the eligibility criteria. Next, the two reviewers evaluated the full texts of the selected publications and independently extracted the following data: author, year of publication, study design, sample size, type of NIBS and its protocol details, EEG acquisition and analysis, and main findings, and inserted the data in a customized table. A search for relevant articles was performed in the reference list of selected articles of the full text. Conflicts were resolved by consensus or by a third reviewer, if necessary. The reviewers involved in the search, screening, and data extraction were previously trained.

Although a quality assessment is not a mandatory stage of the scoping review, previous studies suggest that this is a necessary component in this type of review (26, 27). Since this study reviewed the evidence on the possible neurophysiological effects of a promising treatment for patients with PD, we decided to include the quality assessment of the included studies. We used the modified version of the tool proposed by Donws and Black (28), and with the final score, we classified the studies as “excellent” (24–28 points), “good” (19–23 points), “regular” (14–18 points), or “bad” (< 14 points) (29).

## Results

**Figure 1:**
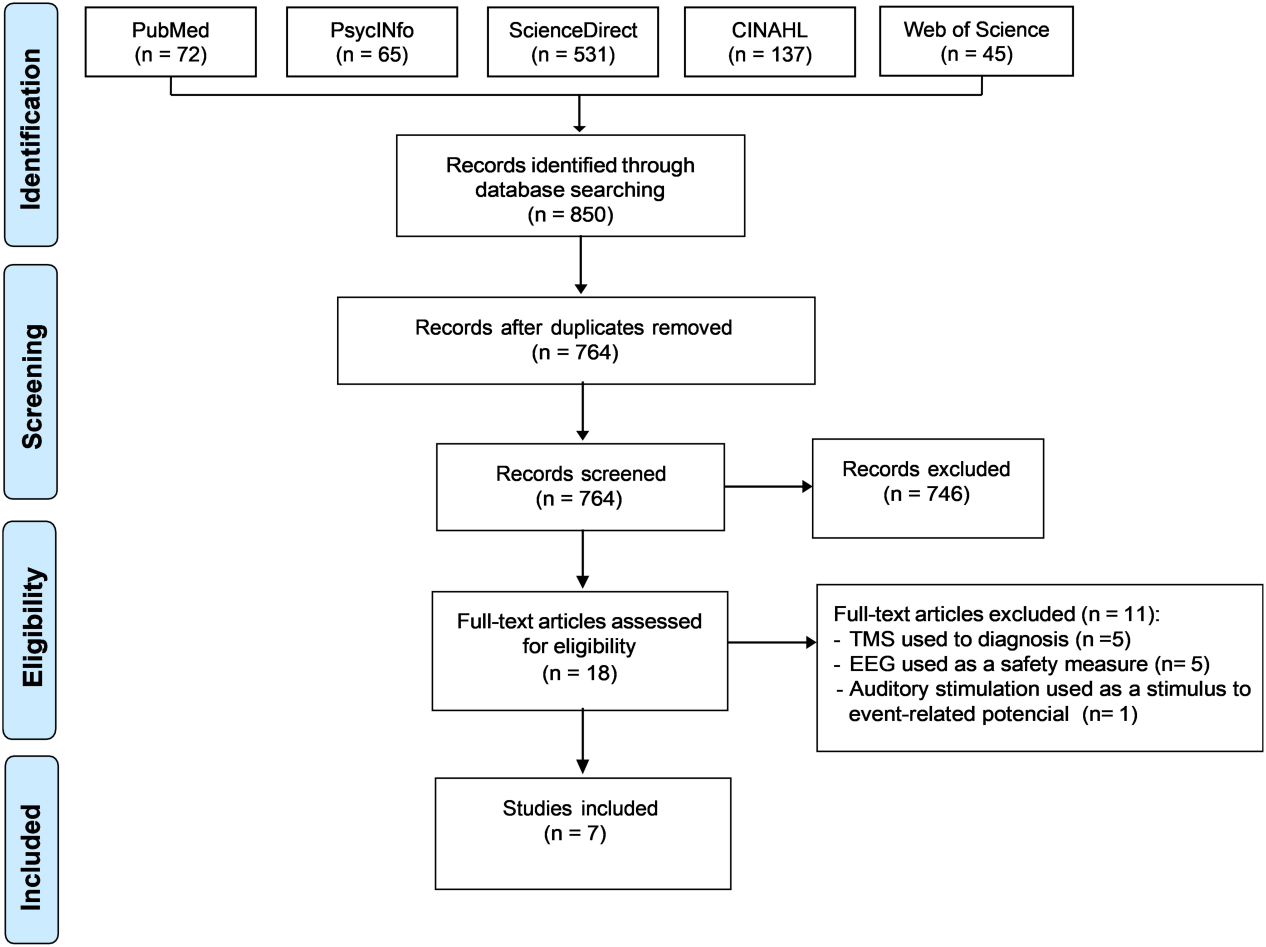
PRISMA flow chart. PRISMA, the *Preferred Reporting Items for Systematic reviews and Meta-Analyses*.

After duplicate removal and screening, seven out of the initial 850 studies were included. The entire search and selection process is pictured in Figure 1. The studies were categorized per NIBS techniques used: TMS (30, 31), tES (32, 33), and other forms of NIBS (34–36).

**Figure 2:**
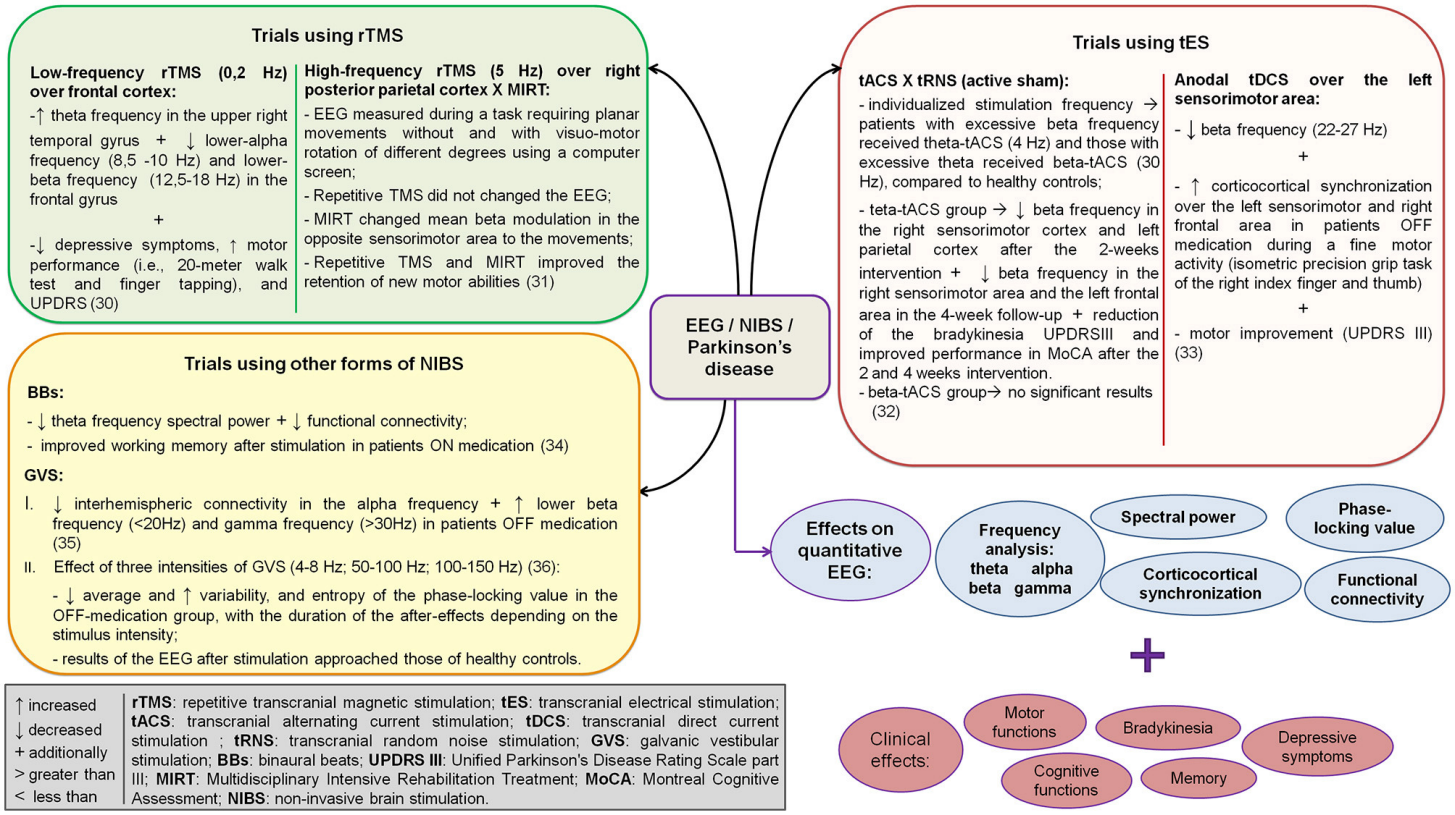
Summary of the main results of the included studies regarding the effects of non-invasive brain stimulation on quantitative EEG and motor and non-motor outcomes in patients with Parkinson's disease. EEG, electroencephalogram.

The main results regarding the effects of NIBS on quantitative EEG and motor and non-motor outcomes in patients with PD are summarized in Figure 2.

### Trials Using Repetitive TMS

Tanaka et al. (30) found increased theta frequency in the upper right temporal gyrus and decreased lower-alpha frequency (8.5–10 Hz) and lower-beta frequency (12.5–18 Hz) in the frontal gyrus after low-frequency (0.2 Hz) rTMS over the frontal cortex (Table 2). These changes in EEG activity were followed by decreased depressive symptoms, improved motor activity (i.e., 20-m walk test and finger tapping), and improved Unified Parkinson's Disease Rating Scale (UPDRS) (30). While Marchesi et al. (31) compared the effects of high-frequency (5 Hz) rTMS to a multidisciplinary intensive rehabilitation treatment (MIRT) on the EEG oscillations of patients with PD during a motor task. They found that despite both techniques improved learning of a rotation task, but only MIRT and not rTMS changed mean beta modulation in the opposite sensorimotor area to the movements, but both interventions improved the retention of new motor abilities.

**Table 2:** Characterization of studies that used transcranial magnetic stimulation in Parkinson's disease.

**References**	**Design: randomization/blinding/sham**	**Sample number (age range in years); sex distribution; stage (disease duration)**	**Stimulation protocol**		**EEG**		**Other outcomes**
			**Type of stimulation; parameters used**	**Number of sessions**	**ON or OFF medication**	**Number of channels; condition of assessment; data analysis**	
Tanaka et al. (30)	No/No/No	7 (66.3); 5 males; HY>2 (NR)	rTMS (0.2 Hz, over frontal areas, 20 times per day, intensity of 1,5 T)	5	ON	20; eyes-closed resting before and after the stimulation; frequency analysis and LORETA	Motor activity with finger tapping and 20-m walking; UPDRS; actigraphy
Marchesi et al. (31)	Yes/No/Yes	29 (60); 23 males; HY 2–3 (8 ± 4 years) + 19 healthy controls (59); 10 males	rTMS (5 Hz, over right posterior parietal cortex)	2 (1 rTMS + 1 sham)	ON	256 (rTMS and control group) and 68 (MIRT group); recorded during motor task; analysis of frequencies calculated in the range of 15–30 Hz (oscillations beta)	Reaction time; amplitude of peak velocity; movement time and extention; directional error; learning and retention

*HY, Hoehn and Yahr Scale; rTMS, repetitive transcranial magnetic stimulation; LORETA, Low-Resolution Electromagnetic Tomography; UPDRS, Unified Parkinson's Disease Rating Scale; MIRT, Multidisciplinary Intensive Rehabilitation Treatment; NR, Not reported*.

### Trials Using tES

The studies that used tES were randomized, blinded, placebo-controlled, and included clinical evaluations of PD. However, the EEG was evaluated during the different status of the parkinsonian medication action, at rest, and during a motor task (Table 3).

**Table 3:** Characteristics of studies with transcranial electric stimulation in Parkinson's disease.

**References**	**Design: randomization/blinding/sham**	**Sample number (age range in years); sex distribution; stage (disease duration)**	**Stimulation protocol**		**EEG**		**Other outcomes**
			**Type of stimulation; parameters used**	**Number of sessions**	**ON or OFF medication**	**Number of channels; condition of assessment; data analysis**	
Del Felice et al. (32)	Yes/Yes/Yes	15 (69); 9 males; HY 1–2 (6.3 ± 4.8 years)	tACS; 4Hz (theta-tACS group) or 30 Hz (beta-tACS group); electrodes over the scalp area in which the power spectral difference was detected and over the ipsilateral mastoid; 1–2 mA, 30 min. -Active sham condition: tRNS alternate current with random amplitude and frequency (1–2 mA; 0–100 Hz), over the same sites of tACS	10 tACS + 10 active sham	ON	32; 10 min of open-eyes resting state, before, immediately after stimulation and at 4-weeks follow-up; analysis of power spectral density and the relative power. -EEG data from 21 healthy controls (45,14 years; 9 males) were used to choose the location and frequency of stimulation	UPDRS III; GDI; frontal-executive functions, memory, and mood
Schoellmann et al. (33)	Yes/Yes/Yes	10 (64.3); 7 males; HY: NR (8.6 ± 4.1 years) + 11 healthy controls (58.6); 6 males	tDCS; over the left sensorimotor (C3, anode) and right frontal areas (Fp2, cathode); 1 mA, 20 min -Sham condition: tDCS with 1 mA discontinued after 40s	2 (1 tDCS + 1 sham)	OFF	25; recorded at rest (3 min.) and during a performance of an isometric motor precision task (3 min.), before, directly after and 30 min after stimulation; analysis of the frequency-domain spectrum (power) and corticocortical connectivity.	UPDRS III (sum of items 22–25, right hand); fine motor assessment

*HY, Hoehn and Yahr Scale; NR, Not reported; tACS, transcranial alternating current stimulation; tDCS, transcranial direct current stimulation; tRNS, transcranial random noise stimulation; UPDRS, Unified Parkinson's Disease Rating Scale; GDI, Gait Dynamic Index*.

Del Felice et al. (32) evaluated the effect of tACS and transcranial random noise stimulation (tRNS), which was used as an active sham, for 2 weeks each in patients with PD. The frequency of stimulation was individualized so that those with excessive beta frequency received theta-tACS (4 Hz) and those with excessive theta received beta-tACS (30 Hz), compared to healthy controls (32). The theta-tACS group presented decreased beta frequency in the right sensorimotor cortex and left parietal cortex after the 2-week intervention and a persistent reduction in the right sensorimotor area and the left frontal area in the 4-week follow-up. The theta-tACS group also improved bradykinesia and performance in the Montreal Cognitive Assessment (MoCA). However, beta-tACS did not yield significant results (32). On the other hand, Schoellman et al. (33) found decreased beta frequency (22–27 Hz) and increased corticocortical synchronization over the left sensorimotor and right frontal area on OFF medication during a fine motor activity after anodal tDCS over the left sensorimotor area. These changes in EEG were accompanied by motor improvement (i.e., UPDRS III) (33).

### Trials Using Other Forms of NIBS

Studies that involved the use of other NIBS were characterized for the use of sham stimulation, similar age, and time of diagnosis of PD between participants. However, although the EEG was evaluated at rest, the studies differed in the condition of eyes open or closed and ON or OFF medication (Table 4).

**Table 4:** Characteristics of studies that used other non-invasive brain stimulation in Parkinson's disease.

**References**	**Design: randomization/blinding/sham**	**Sample number (age range in years); sex distribution; stage (disease duration)**	**Stimulation protocol**		**EEG**		**Other outcomes**
			**Type of stimulation; parameters used**	**Number of sessions**	**ON or OFF medication**	**Number of channels; condition of assessment; data analysis**	
Lee et al. (35)	No/No/Yes	11 (62.1); 4 females; HY: NR (6, 9 years) + 11 healthy controls (59.8); 5 females	nGVS; bilateral and bipolar, over mastoid process, frequency 0.1–10 Hz, during 72 s, followed by a sham current for 60 s	1	OFF	19; eyes open focusing on a fixed target during 60-s pre and post GVS; interhemispheric connectivity analysis (IHC) by Partial Least Squares (PLS) regression and relative contribution percentage	_
Lee et al. (36)	Yes/No/Yes	16 (67.3); 7 males; HY 1–2 (4 ± 4, 3 years) + 18 healthy controls (67.6); 9 males	EVS; bilateral and bipolar, over mastoid process; applied at 90% of the individual threshold level; Three signals in different frequency bands (EVS1: 4–8 Hz; EVS2: 50–100 Hz; EVS3: 100–150 Hz)	4 (Sham, EVS1, EVS2, and EVS3)	ON/OFF	27; eyes open focusing on a fixed target before (20 s), during stimulation (60 s) and after EVS1, EVS2, EVS3 (20 s); analysis of PLV (mean, variability, entropy) and Sparse Discriminant Analysis (SDA)	_
Gálvez et al. (34)	Yes/Yes/Yes	14 (62); 8 females; HY 1–3 (7.2 ± 4, 9 years)	BBs (tones rhythmically at 120 bpm, sinusoidal waveform (154Hz in the left channel and 168Hz in the right channel), which created a 14Hz BB at the brainstem; 10 min. -Control stimulation: BBs without the rhythmically (pink noise); 10 min.	2 (1 BBs + 1 control sound)	ON	29; closed eyes at rest; immediately before and after both stimulations; analysis of power spectral density and functional connectivity	Gait; anxiety; cognition; EKG

*HY, Hoehn and Yahr Scale; NR, Not reported; UPDRS, Unified Parkinson's Disease Rating Scale; EKG, electrocardiogram; PLV, Phase locking value; nGVS, noisy galvanic vestibular stimulation; EVS, electrical vestibular stimulation; BBs, binaural beats*.

Lee et al. (35) found decreased interhemispheric connectivity in the alpha frequency and an increased lower beta (<20 Hz) and gamma (>30 Hz) in PD patients OFF medication after GVS. Lee et al. (36) assessed the effect of three intensities of electrical vestibular stimulation (4–8, 50–100, and 100–150 Hz) and reported decreased average phase locking, increased variability, and entropy of the phase-locking value in the OFF-medication group, with the duration of the after-effects depending on the stimulus intensity. Interestingly, the results of the EEG after stimulation approached those of healthy controls. Finally, Gálvez et al. (34) showed decreased spectral power of the theta frequency, decreased functional connectivity, and improved working memory after a BB compared with the controlled sound in PD patients ON medication.

### Quality Assessment

A single study was classified as presenting excellent methodological quality (32), three as good (33, 34, 36); two as fair (31, 35), and one as poor (30) according to the Downs and Black criteria (Table 5). In general, the studies attended the criteria regarding the reporting section, however, the main factors of confusion in the groups were not listed (30, 35) or were partially listed, and none of the studies mentioned the possible adverse effects of the stimulation. Besides, one of the studies did not present the exact values of probability in the results (30). Some studies did not attend the criteria related to external validity, because few of them reported the location and population of the participants recruited, which does not allow interpretation of the representativeness of the sample (32, 34, 36). Moreover, some studies did not include blinding of participants and personnel (30, 31, 35, 36). Concerning confusion bias/selection, the three studies with the best scores were randomized clinical trials and double-blinded that considered the distribution of factors of confusion in their analysis (32–34). Only one study demonstrated enough power to detect a clinically important effect through power calculations (32).

**Table 5:**
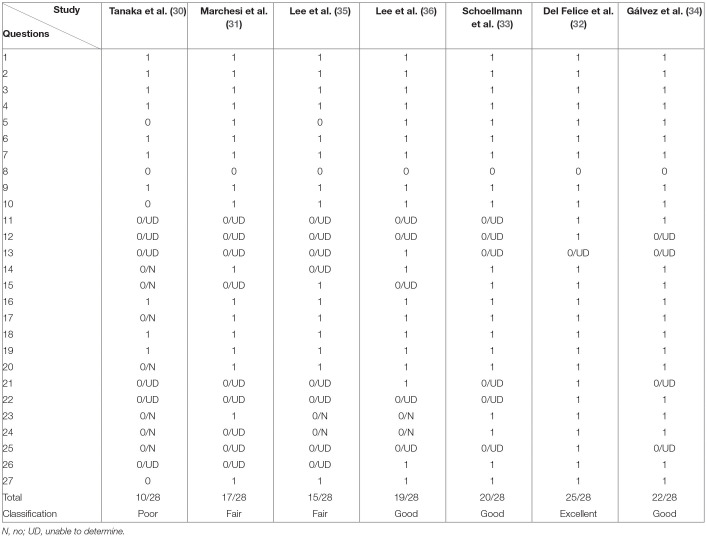
Quality assessment based on the tool proposed by Downs and Black.

*N, no; UD, unable to determine*.

## Discussion

The summary of current evidence suggests that NIBS techniques may change EEG activity, which was associated with improvement in PD symptoms. This scoping review revealed two important findings: (1) there is limited evidence regarding the effects of NIBS on quantitative EEG in patients with PD and (2) the quality of the studies was poor/fair in 3 of the 7 manuscripts based on criteria of Downs and Black.

According to our findings, anodal tDCS, tACS, rTMS, GVS, and BBs consistently showed positive results related to quantitative EEG in the papers reviewed. The majority, but not all the studies, reported clinically significant improvement in patients and a strong relationship between the EEG activity and the movement-related (desynchronization/synchronization), which happens in PD at smaller amplitude (37, 38).

On the other hand, although most studies have shown motor and non-motor improvements that occurred concurrently with changes in the EEG, none of the studies included the analysis of the relationship between EEG at baseline and NIBS-induced changes on clinical outcomes. Additionally, many of the reviewed studies used heterogeneous samples and did not consider possible confounders related to the response rates and adjustments made to control for these variables. Evidence points out that patients with similar clinical characteristics of PD may present different responses to the same treatment, depending on demographic or clinical modifying variables, such as age and disease duration (39, 40). For instance, EEG oscillations have a direct relation in the response to treatment involving synaptic plasticity, thus baseline dysfunction may be also a functional and therapeutic marker for individual and personalized NIBS.

The regions of interest for the treatment of PD varied concerning the type of stimulation and the symptoms treated. Although the NIBS techniques described in these studies have different routes and action mechanisms, all of them aim to induce depolarization mechanisms in an attempt to directly alter brain activity in an extensive neuronal network involved in motor and cognitive processing. It is also important to consider that most of the included studies have consistently failed in detailing the functional impairment of patients which made it difficult to establish a relationship between clinical symptoms and the patterns of the quantitative EEG. PD patients with distinct clinical characteristics could answer differently to excitatory or inhibitory NIBS due to the different brain pattern activation (41). While these results related to aftereffects of NIBS are encouraging, further studies are necessary to elucidate the link between the cortical target, excitatory/inhibitory stimulation, and neural endophenotypes of PD.

It should be noted that all included studies assessed the effects of NIBS on the outcomes in the short term. In fact, the number of sessions ranged from 1 to 10. The study with the longest NIBS intervention and outcome assessment period was of Del Felice et al. (32) with 10 sessions of tACS (over 2 weeks) and outcome assessment at baseline, post-intervention (2 weeks), and 4 weeks after the end of the intervention. They found significant changes in quantitative EEG and improvement in bradykinesia and cognitive performance (32). However, so far, no study has assessed if there would be a significant long-term clinical improvement and quantitative EEG changes. Future long-term trials would greatly advance the current knowledge on this issue since it is difficult to modify a complex dysfunctional network by acute stimulation (42) and it would present important clinical applicability.

The EEG data acquisition protocols varied among studies, concerning the medication status (i.e., ON vs. OFF), “eyes condition” (i.e., closed vs. open), and activity state (i.e., resting-state vs. cognitive/motor tasks). The recording of EEG data and NIBS application during the ON medication may decrease inter- and intra-individual variability. During the OFF-medication motor and/or non-motor PD symptoms appear or are worsened, which are improved after the next dose of levodopa (43). Moreover, studies have shown marked differences in EEG comparing ON and OFF medication in spectral power, coherence, and phase-amplitude coupling (13, 44–46). Hence, when recording EEG, it should be considered that the apparent or intensified motor and non-motor PD symptoms may result in worsened performance, interference in EEG signal, or even data loss (33, 36). For instance, Gálvez et al. (34) calculated the levodopa equivalent dose for each individual and the intervention sessions accompanied by EEG recordings took place on different days, but at the same time of the day to reduce variability due to medication action and time of the day.

Concerning the eyes condition, previous studies were able to differentiate and classify patients with PD and healthy controls at rest with the eyes closed and during tasks with eyes opened (47–50). On the other hand, Railo et al. (51) demonstrated that patients with PD in the initial to intermediate state can be classified with relatively high sensitivity using EEG data recorded at rest with eyes open with about 10 electrodes, located over the motor and occipital areas. Contrary, the classification was not possible with the eyes closed (51). At present, it should be recommended to record EEG both with eyes opened and closed, whenever possible to test if the NIBS-induced changes are detectable at one condition or another or in both conditions.

Concerning the quantitative EEG parameters, the specific parameters measured may depend on the research purpose and study design. For instance, while some studies included in this review have assessed the EEG at rest and analyzed the frequency band spectral power (32), others have assessed the event-related synchronization/desynchronization or corticocortical connectivity during motor tasks (31, 33). Despite strict guidance on quantitative EEG measures to monitor the effects of NIBS may not be provided, future studies should build on previous studies investigating changes in the EEG associated with PD and include at least more common measures used in previous NIBS studies to allow for comparability. For instance, a recent systematic review by Shirahige et al. (52) that includes 19 studies with 312 patients with PD and 277 showed that patients with PD present slower EEG frequencies (i.e., increased slower frequencies and decreased faster frequencies) at rest and during the performance of complex movements. Such results may serve as a starting point to define possible quantitative EEG parameters.

Furthermore, adding EEG measures to predictive models could provide fundamental prognostic value for motor recovery. In this light, the benefit of measuring both white matter tracts integrity and beta oscillatory activity in addition to clinical measures needs to be further explored. Most importantly, computational models could be needed for the design of brain stimulation protocol, considering EEG parameters and individual variability of cortical mapping.

Regarding the quality of the included studies, we identified potential critical bias in different categories. Most of the studies presented no sample size calculation, blinding procedure, and lack of information about the stage of the disease and medication intake dosage. Despite not being clinically representative, these medications can certainly alter treatment outcomes and “mask” the therapeutic effects of these techniques (53).

The main limitation of this systematic review is the heterogeneity of protocols between the included studies could somehow limit our conclusion. Moreover, a high risk of bias is present in several studies, which calls for caution in interpreting the results.

There are multiple sources of potential heterogeneity within the EEG and brain stimulation literature relating to the variability in stimulation parameters and outcomes measured, dose, and clinical characteristics. One of the main factors lacking in half of the studies was robust concordance regarding the enhancement of motor recovery associated with the clinical application of brain stimulation and EEG. Moreover, completeness of evidence is lacking regarding electrophysiological markers reflecting tDCS effects and cognitive outcomes in PD. This is an important factor to take into account when talking about brain modulation techniques and progressive impairment. This diversity of metrics and the lack of clear underlying hypotheses regarding the electrophysiology of motor and cognitive parameters make it hard to interpret the effect of treatment. There is currently insufficient high-quality evidence to make conclusions about the benefits or harms of NIBS and electrophysiologic correlates on PD.

## Conclusion

In this systematic scoping review, current evidence suggests that NIBS could change cortical activity in patients with PD, however, we are not yet able to use the EEG outcomes to predict the cognitive and motor treatment response after brain stimulation. Further studies are also necessary to identify the clinical and neurophysiological optimal parameters associated with NIBS outcomes, taking into consideration these individual cortical pathways. In addition to performing higher quality care of patients. It is important that more funding be directed not only to neuromodulation studies but also to neurobiological studies in PD.

## Data Availability Statement

The original contributions presented in the study are included in the article/supplementary material, further inquiries can be directed to the corresponding author/s.

## Author Contributions

TC participated in conceptualization, methodology, software, and writing—original draft. SS participated in resources and investigation. RS participated in writing and review. DM, SA, and CG participated in writing—review and editing. All authors contributed to the article and approved the submitted version.

## Conflict of Interest

The authors declare that the research was conducted in the absence of any commercial or financial relationships that could be construed as a potential conflict of interest.

## Publisher's Note

All claims expressed in this article are solely those of the authors and do not necessarily represent those of their affiliated organizations, or those of the publisher, the editors and the reviewers. Any product that may be evaluated in this article, or claim that may be made by its manufacturer, is not guaranteed or endorsed by the publisher.
